# Impact of grape (*Vitis vinifera*) seed extract on egg production traits, nutrients digestability, lipid peroxidation and fertility of golden laying hens (*Gallus gallus*) during early stage of production

**DOI:** 10.1080/01652176.2023.2262543

**Published:** 2023-10-04

**Authors:** Abdul Hafeez, Shah Faisal Hassni, Shabana Naz, Rasha Alonaizan, Rasha K. Al-Akeel, Dai Sifa, Shamsuddin Shamsi, Rifat Ullah Khan

**Affiliations:** a Department of Poultry Science, Faculty of Animal Husbandry and Veterinary Sciences, The University of Agriculture, Peshawar, Pakistan; b Department of Zoology, Government College University, Faisalabad, Pakistan; c Department of Zoology, College of Science, King Saud University, Riyadh, Saudi Arabia; d Department of Pharmaceutical and Life Sciences, Jiujiang University, Jiujiang, China; e College of Veterinary Sciences, Faculty of Animal Husbandry and Veterinary Sciences, The University of Agriculture, Peshawar, Pakistan

**Keywords:** Growth, layers, reproduction, egg production, egg quality, grape seed extract

## Abstract

Grape by-products represent outstanding alternatives to replace conventional and unsustainable feed sources, given the substantial quantities generated annually by the winery industry. Regrettably, the majority of these by-products are wasted, resulting in significant environmental and economic repercussions. This study was conducted to assess the growth performance, feed efficiency, egg production and quality, lipid peroxidation, fertility and hatchability of reproductive laying hens during their early production stage. A total of 720 golden laying hens, all approximately 25 weeks old and with similar body weights, were randomly assigned to four experimental treatments (six replicates) as follows: control group receiving only the standard diet, (2) a group receiving the standard diet supplemented with grape seed extract at a rate of 250 g/kg (GSE1), (3) a group receiving the standarddiet supplemented with grape seed extract at a rate of 500 g/kg (GSE2), and (4) a group receiving the standarddiet supplemented with grape seed extract at a rate of 750 g/kg (GSE3). There were no significant change (*p* > 0.05) in feed intak, body weight gain and feed conversion ratio between the control and the experimental groups. Egg weight, egg shell thickness and egg shell weight were significantly (*p* < 0.05) higher in GSE250 GSE500 and GSE750 compared to the control. The results showed that hen day egg production was also significantly higher (*p* < 0.05) in GSE500 and GSE 750 compared to the control. Fertility level of GSE 500 and GSE750 was significantly (*p* < 0.5) higher compared to the control. The MDA level decreased significantly (*p* < 0.05) in the GSE supplemented birds compared to the control. From these findings, we concluded that GSE 750 had positive impact on egg production, reducing lipid peroxidation and improving fertility in golden laying hens.

## Introduction

Scientific literature has documented a keen interest in the use of eco-friendly materials, specifically focusing on the application of medicinal plants and herbal-based extracts (Imran & Alsayeqh 2022; Shehata et al. 2022; Ullah et al. 2022; Aslam et al. 2023; Basiouni et al. 2023). These compounds are rich in bioactive phytochemicals that possess various properties such as anti-inflammatory and anti-inflammatory activities, which have been shown to potentially benefit the reproductive functions (Dadar et al. 2021; Marruchella et al. 2021; Hasan et al. 2022). The production of grapes (*Vitis vinifera sativa*) holds great significance due to the excellent nutritional components and pharmaceutical properties found in is grape berries, both in their raw and dried forms. This is particularly true for grape derivatives like peel and seed extracts. Grape seeds consist of a composition that includes complex carbohydrates (29%), a significant amount of dietary fiber (47%, with a content ranging from 60% to 70%), a notable fat content (13%), a moderate protein content (11%), along with minerals and phenolic compounds (Costa et al. 2022). In addition, grape seeds are composed primarily of proanthocyanidins, with epicatechin, gallic acid, monomeric catechin, polymeric and oligomeric proanthocyanidins being the main constituents (Sun et al. 2018; Chand et al. 2021; Israr et al. 2021; Romero et al. 2022). These compounds have been shown to possess stronger antioxidant properties compared to that of vitamins (Bagchi et al. 2014). While the effects of grape seed extracts (GSE) have been extensively studied in diseases (Olaku et al. 2015; Downing et al. 2017), there is a scarcity of research focused on reproductive efficiency in poultry. Studies conducted on hens have indicated that grape seed proanthocyanidin extract can potentially impede the process of ovarian aging through the mitigation of oxidative stress (Liu et al. 2018). Additionally, it may offer some protection against cadmium endocrine disruptor in reproductive system (Hou et al. 2016). However, no investigations have explored the *in vivo* impact of GSE on egg performance and fertility parameters.

The beneficial effects of antibiotics on animal growth, performance, and health have been extensively documented. However, there is growing concern regarding the misuse and abuse of antibiotics (Moryani et al. 2021), leading to adverse consequences such as the presence of medical residues, antimicrobial resistance, environmental pollution, and potential effects on human health. Consequently, the supplementation of antibiotics for enhanced growth performance in poultry has been prohibited in numerous countries. As a result, there has been a surge in scientific research focused on exploring natural plant-based alternatives that can serve as ‘natural’ additives to enhance animal production performance. In recent years, numerous ‘natural’ additives have been discovered, not only to improve productivity but also to reduce the reliance on antibiotics and mitigate the risk of potential human pathogens (Orinetha et al. 2022).

Egg laying in hens is regulated by the neuroendocrine system. Grape seed extract contains non-­estrogenic or weakly estrogenic flavonoids that enhance estrogenic activity, which act through endocrinal pathwasy to promote follicular maturation and ovulation, resulting in increased egg laying in hens (Sun et al. 2018). Feeding poultry with grape by-products has demonstrated effects that depend on both the dosage and the form of these by-products. Consequently, they are typically integrated into the diet at levels ranging from 6% to 10% (Erinle and Adewole 2022). Most of the studies involving laying hens have been conducted on grape pomace (Goñí et al. 2007; Kara et al. 2016) and little research has been conducted on grpe seed extract (GSE). Previously, Kaya et al. (2014) reported that different levels of GSE improved egg production and antioxidant status of laying hens. Recently, Romero et al. (2022) reported that grape seed extract at the level of 1 g/kg resulted in improvement of only few egg quality parameters but resulted in poor feed intake. No information is avaialble about GSE effects on fertility, hatachability and nutrient digestability potential in laying hens. The objective of the present study was to evaluate the graded levels of GSE on growth performance, egg production and quality, lipid peroxidation and reproductive performance in golden laying hens.

## Materials and methods

### Grape seed extract

Grape seed extract was prepared as described by Kara et al. (2016). Briefly, red grapes (*Vitis vinifera* var. Cencibel) at optimal maturity from the open market were purchased. The grape seeds were then dried in the shade for a period of 3–4 days. After drying, the seeds were powdered in a metallic hammer mill. Approximately 100 grams of the milled seeds were extracted with 500 ml of 100% ethanol at room temperature for 48 h. The supernatants were filtered and the ethanol was subsequently removed from the solution using a rotary evaporator under vacuum conditions at a temperature range of 50–60 °C. The resulting extract was stored at 4 °C in a refrigerator for future use in the study.

### Animals and diets

A total of 720 golden laying hens, all approximately 25 weeks old and with similar body weights, were randomly assigned to four experimental treatments. Each treatment consisted of 180 laying hens, which were further divided into six replicates. Each replicate contained 30 birds housed in two-tier battery cages measuring 35 × 40 × 55 cm. The hens were kept in an open-sided building with an average daily temperature ranging from 25 to 32 °C. The lighting cycle provided to the hens was 16 h of light per day. Nipple drinkers were provided within the cages for water supply. To prevent hens from consuming feed assigned to neighboring replicates, a continuous metal small trough was divided. The trial commenced when the hens reached 25 weeks of age and continued for a duration of 5 weeks, ending at 30 weeks of age. The birds in this experiment were fed a commercial standardcorn-soybean diet, formulated according to the composition outlined in Table 1. The experimental treatments consisted of the following: (1) control group receiving only the standarddiet, (2) a group receiving the standarddiet ­supplemented with grape seed extract at a rate of 250 g/kg (GSE1), (3) a group receiving the standarddiet supplemented with grape seed extract at a rate of 500 g/kg (GSE2), and (4) a group receiving the standarddiet supplemented with grape seed extract at a rate of 750 g/kg (GSE3). Some of the ingredients and their level has been given in Table 2.

**Table 1.** Composition and chemical analysis of standarddiet.

Ingredients	Standarddiet (%)
Corn	55.00
Soybean	22.00
Sunflower meal	5.65
Sunflower oil	7.00
Monocaclium phosphate	3.3
Calcium carbonate	3.4
Vitamin-minerals premix^a^	0.25
L-lysine	0.15
DL-methionine	0.40
Analyzed composition	
Crude protein	17.00
ME (MJ/kg)	11.2^b^
Crude fibre (%)	4.20
Neutral-detergent fibre (%)	12.3
Calcium	2.9
Non-phytate phosphorous	0.55
Methionine-cystine	0.75

^a^ Layer Vit. & Min. premix: consist of VIT E 14 KIU, Vit. A 14 MIU, Vit. B1 1 g, Vit. D3 4 MIU, Vit B6 1.5 g, Vit B2 7 g, Vit B12 10 mg, niacin 28 g, pantothenic acid 9.81 g, biotin 135 mg, folic acid 1.5 g, iron 15 g, copper 6 g, iodine 0.5 g, manganese 65 g, zinc 55 g, selenium 0.14 g and antioxidant 8 g.

^b^ Values calculated from feedstuffs.

**Table 2.** Main ingredients of grape seeds.

Items	Ingredients
Moisture, %	6.57
Crude fat, %	10.12
Crude protein, %	9.35
Neutral detergent fibre, %	42.92
Tocopherol, mg/kg	15.00

### Laying performance

At the start (25 weeks) and at the end of the experiment (30 weeks of age), the birds’ weights were recorded. Daily egg collection was conducted, and egg production was expressed as a percentage of hens-day, representing the number of eggs produced per day. Feed intake was measured weekly on a per-cage basis, considering the total feed consumed by the hens in each cage. The feed conversion ratio (FCR) was determined by dividing the amount of feed consumed by the hens by the total weight of eggs produced. Egg quality traits such as yolk weight, albumin height, albumin weight, Haugh Unit, albumin ratio, yolk and shell ratio were determine. For egg quality evaluation, 30 eggs laid between 08:00 and 18:00 h from each experimental treatment were randomly selected at the end of end of each week and were averaged for the whole experimental period. Individual eggs were subjected to an evaluation of various quality parameters. Using a plate measurement stand known as EQM, the eggs were broken, and the weight of the yolk and the albumen (egg white) were measured. To measure the weight of the eggshell, any albumen adhering to the eggshells was carefully cleaned, and the membrane was subsequently removed. The eggshells were then left to dry at room temperature for a period of 24 h. The weight of the albumin (egg white) and the thickness of the eggshell were measured following the methods described by Saleh et al. (2020). The eggshell thickness was recorded by taking the mean values from three different locations. A micrometer caliper (Mitutoyo, 0.01 to 20 mm, Tokyo, Japan) was used to measure the eggshell thickness after the removal of the eggshell membranes. The Haugh unit was recorded using the following equation as described by Kara et al. (2016)

### Fertility and hatchability measurement

At the end of each week, a total of 20 eggs from each replicate were collected and placed in an incubator for hatching. After the incubation period, the hatched chicks were counted. Any eggs that did not hatch were examined to determine the percentage of fertility (the number of fertile eggs) and hatchability (the number of eggs that successfully hatched) for each replicate as described by Alagawany et al. (2018).

### Plasma melanodialdehyde (MDA) measurement

Blood samples were collected from the 2 birds/replicate for the analysis of plasma MDA. For plasma analysis, blood samples were collected into heparinized tubes. The collected blood samples were then subjected to centrifugation at 700 g for a duration of 20 min in order to separate the plasma from the whole blood. Plasma was stored at −20 C until analysis. Plasma MDA was measured using commercial available kit (Biocheck, USA) according to manufacturer’s guidelines.

### Statistical analysis

The collected data were subjected to statistical analysis using the general linear models procedure in SPSS, following the user’s guide. A one-way analysis of variance (ANOVA) was performed to determine the differences among the treatments. To further evaluate the significant differences, a post hoc test, specifically the Newman-Keuls test, was conducted. The significance level was set at *p* < 0.05 to determine the statistical significance of the observed differences among the treatments.

## Results

Table 3 illustrates the effects of GSE on the feed intake of laying hens fed GSE at different levels. There were no significant difference (*p* > 0.05) in feed intake among all groups with use of GSE. Feed intake at first and second week remained unaffected (*p* > 0.05) among all groups. In 3^rd^ week, feed intake was significant (*p* < 0.05) highly in the control (795 ± 0.46) as against to GSE 750 (791 ± 0.31). Feed intake at week 4 and 5 did not change between the control and treatment groups.

**Table 3.** Effect of supplementation of grape seed extract on feed intake (g) in laying hens during early period.

Groups	Week 1 (g)	Week 2 (g)	Week 3 (g)	Week 4 (g)	Week 5(g)
Control	795	791	795^a^	796	794
GSE250	794	791	794^a^	794	794
GSE500	795	791	794^a^	793	793
GSE750	796	791	791^b^	795	793
SEM	0.22	0.36	0.46	0.50	0.34
P-value	0.217	0.744	0.039	0.059	0.846

Mean values bearing different superscripts in a row differ significantly (*p* < 0.05).

GSE (Grape seed extract) at 250,500 and 750 mg/kg, respectively.

Table 4 demonstrates body weight gain in grams by laying hens at their peak production period fed with grape seed extract at different levels. Weight gain during the entire study period did not change significantly.

**Table 4.** Effect of supplementation of grape seed extract on body weight gain in layer birds during early period.

Groups	Week 1 (g)	Week 2 (g)	Week 3 (g)	Week 4 (g)	Week 5 (g)
Control	25.2	28.6	29.8	25.0	27.7
GSE250	26.5	29.8	28.6	25.0	25.1
GSE500	21.5	25.0	29.0	23.5	25.0
GSE750	28.2	28.0	26.2	28.7	25.0
SEM	1.19	1.68	1.36	1.50	1.45
P-value	0.241	0.807	0.841	0.692	0.907

Means with different superscripts in same row are significantly (*p* < 0.05).

GSE (Grape Seed Extract) at 250, 500 and 750 mg/kg, respectively.

Table 5 presents FCR of laying hens at peak production period fed with different levels of GSE. The results indicated that different levels of GSE did not affect the FCR significantly.

**Table 5.** Effect of supplementation of feed with grape seed extract on feed conversion ratio in laying hens during early period.

Group	Week 1	Week 2	Week 3	Week 4	Week 5
Control	4.80	3.28	2.78	2.15	2.30
GSE250	5.20	3.57	2.58	2.69	2.14
GSE500	4.69	3.39	2.54	2.50	2.14
GSE750	4.71	3.47	2.57	2.43	2.04
SEM	0.12	0.11	0.05	0.07	0.03
*P*-value	0.460	0.846	0.404	0.091	0.061

GSE (Grape seed extract) at 250,500 and 750 mg/kg, respectively.

Table 6 shows of supplementation of GSE on egg weight, shell thickness, yolk weight, albumin weight and Haugh Unit in laying hens during the early stage of production. Egg weight, egg shell weight and egg shell thickness were significantly (*p* < 0.05) higher in GSE 500 and GSE 750 compared to the control and GSE250. No significant changes were noted in the other parameters.

**Table 6.** Effect of supplementation of grape seed extract on egg weight, shell thickness, yolk weight, albumin weight and Haugh Unit in laying hens.

Groups	Egg weight (g)	Eggshell Thick (mm)	Yolk Weight (g)	Shell weight (g)	Albumin Weight (g)	Haugh Unit
Control	51.98^b^	0.321^b^	14.5	4.55^b^	30.6	86.5
GSE250	51.31^b^	0.324^b^	15.0	4.91^b^	31.0	86.2
GSE500	54.61^a^	0.329^a^	15.92	4.99^a^	30.5	85.4
GSE750	55.01^a^	0.330^a^	14.91	4.90^a^	30.2	86.3
SEM	0.213	0.001	0.03	0.11	0.11	0.22
*P*-value	0.04	0.04	0.88	0.046	0.55	0.54

Mean values bearing different superscript in a column differ significantly (*p* < 0.05).

GSE (Grape seed extract) at 250,500 and 750 mg/kg, respectively.

Table 7 shows the effect of supplementation of GSE on reproductive performance and MDA level in laying hens. The results showed that hen day egg production was significantly higher (*p* < 0.05) in GSE500 and GSE 750 compared to the control and GSE250. Fertility level of GSE 500 and GSE750 was significantly (*p* < 0.5) higher compared to the control and GSE250. The MDA level was significantly (*p* < 0.05) higher in GSE750 compared to the control.

**Table 7.** Effect of supplementation of grape seed extract on reproductive performance and MDA level in laying hens.

Groups	Hen Day egg production (%)	Fertility (%)	Hatchability (%) (from the total set of eggs)	Hatchability (%) (from the fertile eggs)	MDA (nmol/ml)
Control	66.5^c^	85^b^	79	76	2.81^a^
GSE250	65.8^b^	88^b^	77	76	2.61^b^
GSE500	68.3^a^	90^a^	78	80	2.67^ab^
GSE750	69.6^a^	92^a^	83	81	2.62^b^
SEM	0.97	3.50	1.89	1.30	0.12
P-value	0.61	0.04	0.66	0.61	0.023

Means with different superscripts in same column are significantly different (*p* < 0.05).

GSE (Grape seed extract) at 250,500 and 750 mg/kg, respectively. MDA: melanodialdehyde.

Table 8 shows the nutreints digestability in birds in response to supplementation of GSE at early production period in laying hens. The results showed that there was no significant difference in the parameters of nutrient digestability between the control and treatment groups.

**Table 8.** Effect of supplementation of feed with grape seed extract on apparent total digestibility of nutrients (%) at early production period in laying hens.

Groups	DM (%)	Ash (%)	CP (%)	C Fat (%)	NFE (%)	C Fiber (%)
Control	72.3	48.2	78.3	80.5	72.3	42.2
GSE250	71.8	48.3	78.4	80.6	73.1	42.3
GSE500	72.6	48.3	78.5	80.9	72.5	42.8
GSE750	72.3	48.5	78.4	80.7	72.5	42.5
SEM	0.11	0.04	0.04	0.11	0.12	0.10
P-value	0.107	0.163	0.376	0.695	0.068	0.147

GSE (Grape seed extract) at 250,500 and 750 mg/kg, respectively.

## Discussion

In the current study, feed intake, body weight gain and FCR did not change signficantly after GSE dietary supplementation at 250–750 g/kg. Sayago-Ayerdi et al. (2009) concluded that grape seed byproduct included over 6% in the feed has a negative effect on the feed intake. Other previous research has reported zero or negative effects of grape byproducts in animal diets on feed intake (Chamorro et al. 2013; Romero et al. 2021). These effects are often attributed to the high concentrations of grape polyphenols, specifically condensed tannins, present in these byproducts (Romero et al. 2022). These condensed tannins have been found to bind to proteins, leading to a reduction in protein digestibility. The hindered protein digestion can subsequently result in detrimental effects on the productive performance of animals (Sun et al. 2018). These findings highlight the importance of considering the presence of condensed tannins and their potential impact on protein digestion when incorporating grape byproducts into animal diets. It seems that at the inclusion levels of GSE used in the present study no significant difference in the growth parameters were observed probably because these levels were not high enough to produce any significant change. At the same time, the previous researchers have mostly used grape pomace which is different from GSE used in the present study in term of biochemical composition.

In the present study, hen day egg production, egg weight, egg shell thickness and egg shell weight increased significantly in the GSE 500 and GSE 750 groups. The process of egg laying in hens is a complex physiological process regulated by the neuroendocrine system. Grape seed extract possesses non-estrogenic or weakly estrogenic in nature, which exert their stimulating effect by binding to the same site as steroidal estrogens. This binding enhances estrogenic activity and increases estrogen and progesterone levels in the body. Estrogen and progesterone, working together, stimulate the pituitary gland to release FSH and LH. The presence of FSH and LH promotes follicular maturation and ovulation, leading to increased egg laying in hens (Sun et al. 2018). The mechanisms involving estrogen and progesterone, as well as the release of FSH and LH, play vital roles in the regulation of the reproductive process and ultimately contribute to egg production. In the present study, egg quality parameters such as egg weight, egg shell weight and thickness were significantly improved in the supplemented groups especially GSE 500 and GSE 750. Barbe et al. (2020) found that 1% GSE resulted in improvement in egg weight at the 33 weeks of age. Similar to our findings, Kaya et al. (2014) reported that addition of GSE linearly increased egg production in laying hens. Some authors have attributed the better egg quality to the presence of proanthocyanidin in the grape seeds which have resveratrol like action (Sahin et al. 2010; Barbe et al. 2020). Other authors have linked the height egg weight to the probiotic like effect of grape seeds (Kara et al. 2016).

In the current study the blood MDA significantly decreased in the experimental groups. Kara et al. (2016) found that plasma MDA concentration decreased in laying hens. Kaya et al. (2014) and Barbe et al. (2020) reported that GSE supplementation reduced free radicals formation in egg yolk of laying hens. According to Brenes et al. (2010), the antioxidant activity observed in GSE is associated with its total phenolic content. GSE, which is rich in polyphenols, can serve as an effective source of antioxidants in chicken diets. This suggests that incorporating GSE into the diet of chickens can provide beneficial antioxidant effects. Hajati et al. (2014) demonstrated that GSE supplementation can indeed enhance hatchability. The improved fertility results could be due to the antioxidant effect of GSE in the present study. Genetic selection and unrestricted feeding result in an excessive intake of energy, which leads to abnormal follicular recruitment and disruption of the selection of preovulatory follicles. Consequently, this triggers multiple ovulations and reduces reproductive efficiency. This decline in reproductive efficiency is associated with elevated levels of oxidative stress in animals. To enhance fertility, dietary supplementation strategies incorporating antioxidants have been employed. According to Grandhaye et al. (2021), the supplementation of synthetic antioxidants like vitamin E and selenium has been shown to enhance fertility in laying hens. Therefore, based on the decreased MDA activity observed in our current study, we can infer that the antioxidative activity is responsible for the improved fertility in laying hens.

In this study, no significant difference was found on apparent nutrients digestability in the control and treatment groups. To the best our knowledge, little is available in the published literature on the effect of GSE on the nutrients digestability in poultry. Previously, Goñí et al. (2007) reported that inclusion of grape pomace at the levels of 5, 15 and 30 g/kg did not affect apparent ileal digestability of protein and amino acids, which was attributed to the low contents of polyphenols in the experimental diets. In the study of Aditya et al. (2018) only ash content among the apparent total tract digestability was improved in a quadratic manner in broilers fed with 5 to 10 g/kg grape pomace. However, further research is needed to explore the effects of GSE on the nutrients digestability in poultry.

## Conclusion

From these findings, we concluded that GSE 750 had positive impact on egg production and reducing lipid peroxidation and improving fertility in golden laying hens.

## Data Availability

On request.
